# Crisis management, surveillance, and digital ethics in the COVID‐19 era

**DOI:** 10.1111/1468-5973.12398

**Published:** 2022-02-07

**Authors:** Kees Boersma, Monika Büscher, Chiara Fonio

**Affiliations:** ^1^ Department of Organization Sciences, Faculty of Social Sciences, Faculty of Science, Section of Science, Business and Innovation Vrije Universiteit Amsterdam Amsterdam Netherlands; ^2^ Department of Sociology, Centre for Mobilities Research Lancaster University Lancaster UK; ^3^ Departement of Organization Sciences Vrije Universiteit Amsterdam Netherlands

**Keywords:** crisis management, digital ethics, surveillance

## Abstract

In this special issue, we reflect on the global coronavirus disease 2019 (COVID‐19) crisis and the containment measures put in place by formal authorities, combining both theoretically and empirically three different fields of study: crisis management, surveillance studies, and digital ethics. The special issue shows how the intersection of these fields provides a great opportunity to better understand challenges that are of critical importance to today's societies, as well as opening up new avenues for innovation. The focus of this special issue is to unpack and understand the debate on crisis management measures, surveillance, and ethical consequences during the ongoing, enduring COVID‐19 crisis. Building on crisis management literature, surveillance studies, and digital ethics research the articles included in this special issue reflect on issues of governance, space, as well as moral and ethical considerations, which were often overlooked in the public discourse in relation to the COVID‐19 pandemic. The special issue provides a deeper and clearer understanding of intended and unintended ethical and political consequences of crisis management practices, such as a politics of visibility that makes the operation of power invisible and fails to combat inequality, whilst ignoring the potential positive power of digital data and surveillance for empowerment and resilience

1

In this special issue, we reflect on the global coronavirus disease 2019 (COVID‐19) crisis and the containment measures put in place by formal authorities, combining both theoretically and empirically three different fields of study: crisis management, surveillance studies, and digital ethics. We show how the intersection of these fields provides a great opportunity to better understand challenges that are of critical importance to today's societies, as well as opening up new avenues for innovation (Boersma & Fonio, 2018, Perng et al., 2021). This is a distinctly interdisciplinary effort.

Crisis management studies bring together researchers in disaster sociology, management studies, psychology, geography, and other disciplines to examine the organizational and political governance challenges that crises bring (Boin et al., 2020). Inspired by Perrow's book *Normal Accidents* (1984) and Ulrich Beck's *Risk Society* (1989), crisis management researchers recognize the need to ‘improve our understanding of crisis management processes; how to effectively plan for crises, act during them, and learn from these episodes’ (Deverell, 2012). But many crisis management innovations have increased surveillance of citizens and responders alike, using location, health, and many other forms of data, amounting to a form of “datafication” (van Dijck, 2014) of crises. Surveillance studies scholars have shown that systematic collection of data for crisis management may provide some certainty and a sense of control, but also create “perilous transparency” (French & Monahan, 2020; Monahan, 2021). Surveillance studies leverage insights from philosophies of governance from Carl Schmitt to Foucault to develop critiques of the social and societal consequences (see Adey et al., 2015 for an overview). Most importantly, surveillance in crises enables new forms of social sorting and discrimination (Boersma & Fonio, 2018).

But these consequences are not inevitable, and the growing role of digital technologies in crisis management and surveillance has inspired a turn to digital ethics. Here, researchers study public perceptions, values, and intended and unintended consequences of digital innovation (Floridi et al., 2019). The age of big data has brought considerable public disquiet about the use of personal data even in efforts to control communicable diseases (Gilbert et al., 2019). Concerns often focus on the need to safeguard democratic and humanitarian values, from dignity and freedom to equality and nondiscrimination. Research can be normative, providing, for example, ethics and governance guidance for digital contact tracing for pandemic response (Kahn, 2020), as well as explore metaethical questions. For example, Morozov (2013) challenges the simplistic “solutionism” that characterizes some forms of digital innovation, and the European Data Protection Supervisor's academic Ethics Advisory Group examines foundational values as well as socio‐cultural shifts such as the emergence of the digital subject and the move from a risk to a “scored” society (2018). Attention to the ethics of datafication in crises has prompted a turn to approaches of ethics‐as‐accompaniment (Verbeek, 2011, 2020), and ethical impact assessment (Büscher et al., 2018).

The COVID‐19 global crisis has been framed as a high impact and slow burning, creeping crisis (Boin et al., 2020, 2021). It has disrupted societies around the globe in unprecedented ways, challenging social and political infrastructures, and it has given rise to unprecedented digital surveillance. Digital technologies such as health codes, temperature sensors, track and trace apps, have huge potential to support COVID pandemic crisis management, but they also intensify aspects of surveillance. Severe measures such as lockdowns, social distancing, testing, and contact tracing have been put in place globally in response to the pandemic. These containment measures are slowing down the spread of the virus, but an unfortunate consequence is that some measure expose citizens to invasive surveillance (Eck & Hatz, 2020), and societies to erosion of civic liberties and values, as well as deepening inequalities (Everts, 2020; Kitchin, 2020). The contributions to this special issue explore examples from different countries and with different perspectives.

What makes slow‐burning crises particularly difficult to deal with is that they (and the meanings attached to them) can change over time. The slow‐burning COVID‐19 crisis can be seen as a dynamic, nonlinear problem (Rothan & Byrareddy, 2020), and because there are no linear solutions to this nonlinear problem, the measures taken will have uncertain effects for the long run, and create a long‐lasting impact on societies worldwide. By bringing crisis management, security, and digital ethics research together, we can deepen our understanding of the complex surveillance‐related challenges and opportunities that arise in this context.

Governments, authorities, and crisis management organizations were expected to “fight” the crisis and–often using the rhetoric of war–tried to get the situation back to some sort of “normal.” This involved putting digital surveillance technologies in place that are intrusive and–often as an unintended consequence–not only have a huge impact on individuals' personal lives, constraining mobility, ability to work and earn a living, and social contacts, but also engender more wide‐ranging societal consequences. The surge in digital surveillance has legitimized and normalized personal data collection (Ausma Bernot and Marcella Siqueira Cassiano, this issue) and the exclusion and invisibilisation of, for example, communities in deprived urban areas, the homeless, and undocumented migrants, undermining “trust and solidarity, agency, transparency along with the rights and values of citizens” (Isaac Oluoch, this issue. Mainly, but perhaps not always exclusively for the sake of public health, some emergency measures can remain in place for a long time, contributing to already well‐equipped “surveillance societies” (Lyon, 2001). Moreover, this datafication of disasters can intensify the extraction and exploitation of personal data for commercial and political gain, what Shoshana Zuboff (2019) describes as “surveillance capitalism.”

Zuboff argues that surveillance capitalism is “as significant a threat to human nature in the twenty‐first century as industrial capitalism was [and is] to the natural world” (Zuboff, 2019:v). Zuboff's warnings pre‐date the pandemic, and while COVID control measures may have intensified it, surveillance capitalism was well underway before 2020. Zuboff argues that it is characterized by increased investments in bureaucracies and techniques to systematically–and over longer time‐periods–collect, store and use information for the purpose of controlling behaviours and situations. In this regard, the COVID‐19 pandemic, like other crisis situations, can be seen as a policy window in which advocates see the opportunity to define a problem as “ripe” for surveillance solutions they already have at hand (Boersma et al., 2014; Do Carmo Barriga et al., 2020; Wagenaar & Boersma, 2008). This allows a problematic confluence of surveillance with “disaster capitalism,” described by Naomi Klein as “orchestrated raids on the public sphere, combined with the treatment of disasters as exciting market opportunities” (Klein, 2008:6; Perng et al., 2021).

Digital ethics research provides insight into some of the challenges and opportunities arising at this juncture. Concerns with ethics have accompanied digital technology development from its inception (Floridi, 1999; Weizenbaum, 1976; Wiener, 1950), and European scholars, in particular, have built on foundational values of dignity, freedom, autonomy, solidarity, equality, democracy, justice, and trust to develop a framework that sees digital innovation as an inherently ethical process and a key part of responsible research and innovation (EDPS European Data Protection Supervisor, 2018; Floridi et al., 2019). This recognizes that the design and use of digital technologies is deeply entangled with society and that digital ethics cannot simply tell designers and users what (not) to do. Instead, attention to ethics has to anticipate and evaluate intended and unintended wider societal consequences and be part of the innovation process, as an accompaniment (Verbeek, 2011). It must be focused on articulating critique as well as constructive responses to complex challenges. The Expert Ethics Advisory Group to the European Data Protection Supervisor, for example, highlights that the new European General Data Protection Regulation (GDPR) stipulates purpose binding in ways that “may be at odds with some premises and applications of big data,” such as discovery of invisible patterns in large collections of data (EDPS European Data Protection Supervisor, 2018:7). The challenge of digital ethics research is to drive the ambition of socio‐technical innovation to address such complex contradictions. This may include normative endeavours such as value‐sensitive design (Friedman et al., 2006) and creative ethical impact assessment (Büscher et al., 2018) as well as more metaethical arguments, for example about the inherent “solutionism” of technocratic responses to complex problems (Morozov, 2013).

Particularly exciting recent developments in the field of digital ethics include Timothy Wu's critique of corporate power over digital platforms (Wu, 2018) and Morozov and Bria's (2018) work on promoting, finding, and designing open alternatives to black‐boxed corporate platforms and algorithms for the development of smart cities. Their research is truly transformative at a practical and policy level, introducing real possibilities for more responsible and circumspect digital innovation. For example, Francesca Bria was Chief Digital Technology and Innovation Officer for the City of Barcelona and she is now President of the Italian National Innovation Fund to develop open platform digital strategies and support for citizens' data sovereignty, while Timothy Wu has been appointed to US President Biden's National Economic Council to develop antimonopoly policies.

In this context, the COVID‐19 crisis should not be considered an isolated crisis, as the climate crisis further challenges notions of “normal” at an even deeper existential level. As far as crisis management is concerned, Malm observes that the COVID‐19 and the climate change crises have provoked antithetical reactions (Malm, 2020). While the COVID crisis has triggered drastic measures on a global scale, the climate crisis still suffers from discourses of delay (Lamb et al., 2020). But the burgeoning technocratic promotion of surveillance for smart city sustainability puts deeper datafication of everyday life on the horizon, which is inspiring intensive investment in sustainable smart city surveillance as well as surveillance of eco protest movements. Bringing crisis management research, surveillance studies, and digital ethics considerations arising from the COVID crisis together in this special issue can inform crisis policymakers, crisis management practitioners, activists, citizens, and designers of crisis management and surveillance technologies as they prepare for ethical challenges and opportunities arising in crisis management responses to the climate crisis.

Digital surveillance measures to combat COVID‐19 have been framed as important conditions under which societies can “re‐open” again, allowing for a loosening of lockdowns, despite the fact that the effectiveness of some of those measures (e.g., contact tracing apps) remains unclear and/or would require more comprehensive assessments (Grekousis & Liu, 2021; Kitchin, 2020). In this way, the COVID‐19 crisis provides legitimation for authorities, often in coalition with the private sector, to use existing and collect new citizens' data on a large scale (including mobility, contact, health, and social media data). A “Schmittian” emergency discourse perpetuates the false justification that extensive surveillance is a necessary “trade‐off” between public health and security in exchange for a certain loss of privacy and civil liberties (Kerr, 2008; Kitchin, 2020).

The implementation of surveillance techniques may be done with genuine intentions of care, but still produce deleterious societal consequences. Such societal consequences occur when citizens do not raise the alarm and where there is no push‐back at a local level or a political level. China is an example of where the push‐back is not occurring openly but in other parts of the globe it certainly does take place (Do Carmo Barriga et al., 2020). Yet, in the heat of the struggle of getting the pandemic under control the dark side of surveillance is easily overlooked (Couch et al., 2020; Ram & Gray, 2020). However, a careful debate is urgently needed: surveillance in the COVID‐19 crisis management also needs to be examined as a political process involving questions of power, accountability, and transparency, especially in the face of the even larger unfolding crisis of climate change. Theoretical and empirical studies of surveillance in crises have revealed important aspects of how the intersection of surveillance and crisis can affect social practices and social norms, as well as modes of governance and societal cohesion. Surveillance studies have highlighted wide‐ranging effects of “datafication” on individuals and society, including investigations of global data flows, political interdependencies, and dynamics (Van Dijck, 2014), as well as the effects of surveillance society and surveillance capitalism on civil liberties, civic values and human nature (Lyon, 2001, Zuboff, 2019).

As argued by Martin (2021) in relation to Aadhar, the world's largest biometric identification system, “these trends grew before 2020, but the COVID‐19 pandemic has provided advocates of digital identity with a new crisis through which to promote and legitimize identification systems, particularly in low and middle‐income countries.” While ethical considerations as well the governance of digital technologies existed before the pandemics, the growing reliance of digital technology to tackle crises has exacerbated the need to address the problems arising from this datafication of crises (Taddeo, 2020). Furthermore, surveillance for public health purposes–indirectly–led to an increase in surveillance in other areas, for instance, surveillance of employees working from home, and commercial mental health monitoring apps (Cosgrove et al., 2020). It should also be considered that COVID‐19 related surveillance measures did not happen in a vacuum and will not evaporate after the crisis. Major crises can in fact habituate people to, and normalize, mass surveillance, as in the case of 9/11 in the United States (Pilkinton, 2021).

Mass surveillance is often triggered by a command‐and‐control‐style of governing during crises. However, a high degree of centralization in governing the pandemics, for instance in the UK, did not lead–especially in the first wave of the virus–to increased capacity to manage the COVID‐19 crisis in an effective way (Joyce, 2021). Hence, from crisis management, surveillance studies, and digital ethics perspective, the use of surveillance in global crises like COVID requires in‐depth analyses at the intersections of several concerns, domains, and topics including risk assessments, mode of crisis management as well as both centralized and less centralized approaches, intended and unintended consequences and ethical implications that increasingly draw on surveillance measures.

This special issue argues that transdisciplinary approaches can and must take the debate on surveillance and crises management beyond calls for a more balanced trade‐off of privacy for security, and open it up to a wider consideration of digital ethics. Surveillance studies critiques have shown time and time again how problematic and wrong‐footed discourses of “inevitable” trade‐off are. Introna (2007), for example, reveals that a denial of the fundamental entanglement of the social and the technical is at the heart of platform power. Trade‐off discourse artificially separates “technical means” from “social ends” to divide and rule, and to claim as common sense a logic of causal inevitability. Solove (2011) shows that this inevitability is false, as there are many ways to balance privacy and security by “placing security programs under oversight, limiting future uses of personal data, and ensuring that programs are carried out in a balanced and controlled manner” (p. 207). Most recently and specifically related to the COVID‐19 pandemic, Kitchin (2020) reinforces these arguments, highlighting how the discourse of trade‐off is rooted in technological solutionism, where public education, voluntary measures, and compliance would often be more effective to support smart societies to have both, privacy and public health. At this juncture of critique lies a unique opportunity to open up the debate further to a more nuanced understanding of both challenges and opportunities for the use of personal data in crises.

The focus of this special issue is to unpack and understand the debate on crisis management measures, surveillance, and ethical consequences during the ongoing, enduring COVID‐19 crisis. Building on crisis management literature, surveillance studies, and digital ethics research we seek to contribute to a deeper understanding of surveillance in crises. The articles included in this special issue draw on a wide variety of disciplines and approaches that allow for more comprehensive reflections on issues of governance, space, as well as moral and ethical considerations which were often overlooked in the public discourse in relation to the fight against the COVID‐19 pandemic. The contributions examine key issues that emerged in China, low to middle‐income countries such as the Philippines, Brazil or South Africa, and Europe to enrich a pan‐global debate, drawing together insights from the fields of crisis management, surveillance studies, and digital ethics research. They specifically explore two dimensions:

1. Governance and spatial logistics of containment.
2. Socio‐technical and ethical dimensions of surveillance technologies.

The governance angle emerges, inter alia, in the first article of this special issue, written by Ausma Bernot and Marcella Siqueira Cassiano. Drawing on documentary analysis, they reflect on China's COVID‐19 pandemic response focusing on socio‐political, technological, and psychological perspectives. The authors analyze the complex Chinese pandemic infrastructure, highlighting how ‘Chinese state bureaucracy has used the pandemic to recreate, legitimize, and strengthen its governance apparatus, particularly surveillance technologies’.

While many analyses of China's use of digital technologies focus on the coercive aspects of authoritarian surveillance, Bernot and Siqueira Cassiano show that the realities of COVID surveillance in China are complex and ethically ambiguous. For example, the focus on collective well‐being, family, voluntary compliance, and the linking of individual‐level data supported by neighbourhood “grid” surveillance introduces a degree of participation, agency, solidarity, and responsibility. Encouragement of “mutual surveillance” is a deeply disturbing concept for western political sensitivities shaped by experiences of neighbourhood surveillance under Germany's oppressive and murderous National Socialist government (1933–1945), but it also paves the way for “social surveillance” of the government's response to disasters through social media, allowing citizens to hold the government to account (Noesselt, 2014). And a focus on mental health surveillance and service provision reveals a strong element of (paternalistic) “care” at the heart of the Chinese approach to COVID surveillance (c.f. Kim et al., 2021, for a feminist critique of the notion of care through surveillance).

This should, of course, in no way dilute critiques of China's and other authoritarian governments' misuse of surveillance data. Human Rights Watch (2020) alerts about the abuse of power and discrimination against minorities in China's response to COVID are deeply concerning. However, consideration of the complexities and ambiguities of ethical and political practices of surveillance under authoritarian governance also reveals how Chinese authorities are struggling to govern richly datafied, interconnected, and social media savvy populations of digital “doing subjects” (Ding, 2020; Ruppert et al., 2013; Tyfield, 2017). Western democratic governments experience different challenges such as fake news and post‐politics, with Mayer‐Schönberger citing the 2016 Trump elections and the Cambridge Analytica scandal to argue that digital “platforms have become weaponized to unravel not just privacy, but the very fabric of democracy” (2021:1). But exploring and comparing the complexities, unintended consequences, and the ethical and political ambiguities of surveillance in different countries and political contexts can move knowledge and debates onto another level. There are many opportunities for learning.

Perhaps one of the most disturbing insights Bernot and Siqueira Cassiano provide is the Chinese government's focus on mental health in crisis situations. They argue that “the most important legacy of China's psychological response to the pandemic refers to the transformation of mental health into a site of governance.” The European Data Protection Supervisors' Expert Advisory Group on digital ethics pointed out in 2018 that one of the biggest ethical challenges for democratic societies is the move “from governance by institutions to governmentality through data” (p. 12), because it allows nudging and even manipulation for commercial and political purposes, the lifeblood of surveillance capitalism, to flourish. The 2016 Cambridge Analytica scandal and the more recent disclosures that Facebook is deliberately fueling outrage,^1^ makes the fact that the Chinese government is targeting citizens who may be fearful, anxious, angry, or disappointed with mental health measures seem like a route towards pathologizing dissent, a disturbing violation of digital ethics, with clear resonances beyond China's authoritarian regime.

In their contribution to this special issue, Eula Bianca J. Villar and John Pascual Magnawa explicitly explore “inappropriate surveillance” effects and implications of COVID‐19 surveillance in countries with a proclivity for populism, as well as resource constraints and known issues with poor governance, with a specific focus on the Philippines. The governance perspective allows them to shed light on how emergency management that falls short of addressing the population's needs, prompts actors to self‐help, which further weakens already fragmented crisis governance approaches. Villar and Magnawa, too, observe ethical consequences, including erosion of civil liberties and human rights violations, a militarization of crisis governance, and the spread of divisive narratives. Specifically, their analysis highlights the inequalities nested within regions and countries, and how these are exacerbated by surveillance under rising populism. The pandemic has been used to increase algorithmic categorization and “heavy policing to create a clear depiction of [and discrimination against] ‘perpetual enemies of health and order,’” making “public examples of their [the state and the police's] capability to arrest violators of the quarantine protocols,” hitting disadvantaged and vulnerable populations in deprived urban areas and the informal economy the hardest, as well as “individuals who actively support human rights advocacies.” Bringing such analysis from non‐European contexts to the table is highly illuminating, highlighting not least of all, the fragility of democratic institutions, conventions, and values.

Turning attention to the far more affluent and model democracy conditions in Switzerland, Francisco Klauser and Dennis Pauschinger explore how there, too, COVID surveillance is linked not only to curtailment of freedom of movement for public health, but also “social sorting and loss of informational freedom.” To examine the spatialities of control and surveillance, Klauser and Pauschinger draw upon existing literature on the containment of pandemic disease in general while also referring more specifically to examples on the fight to COVID‐19 in Switzerland. He examines three spatial logics of control: border and access, the monitoring of people and objects, and the internal organization and monitoring of spatial enclaves. Klauser and Pauschinger invite a more sustained research agenda that has as its core issue of power and deals with the policy‐making, the executive, and the citizen levels. This is a highly timely call for more attention to pandemic biopolitics through mobility. As Lorenzini (2020:S43) points out, biopolitics is “crucially, a matter of governing mobility–and immobility.” Making the link between the COVID pandemic and other crises, including the climate emergency, Lorenzini asks whether the experience of COVID surveillance.

*Will help us realize that the ordinary way in which “borders” are more or less porous for people of different colors, nationalities, and social extractions deserves to be considered as one of the main forms in which power is exercised in our contemporary world (ibid)*.

Klauser and Pauschinger trace the spatial and mobility politics of pandemic surveillance, arguing that scrutiny of the digital ethics of social sorting, informational freedom, and digitized mobility management must be a critical part of investigations of social and spatial justice.

Such concerns with justice draw attention to how “biopolitics is always a politics of *differential vulnerability*” (Lorenzini, 2020:S43), which brings challenges as well as opportunities. Isaac Oluoch's contribution to this special issue is an analysis of “vectors of vulnerability” in deprived urban areas (slums, favelas, and informal settlements). He explores the ethical, moral, and political dangers and transformative potential of geoinformation for managing risk and governmentality. He studies how individuals and communities experiencing marginality are often in a state of “permanent emergency,” citing Bankoff (2004), and how having to cope with COVID has added complexity. The use of geoinformation in COVID crisis management can endanger “ethical values such as trust and solidarity, agency, transparency along with the rights and values of citizens.” For example, maps are not objective representations of reality. It really matters “*who* is doing the representing and mapping,” because “maps are arguments about existence” (Wood et al., 2010:34, cited in Oluoch, this volume). Many deprived urban areas are not mapped, and as such, they are made invisible and often not considered for support. They are, however, very much considered when it comes to controlling mobility, as we also saw in Villar and Magnawa's findings of increased surveillance, policing, and militarization of COVID control in the Philippines.

Oluoch examines the mapping practices that underpin–and challenge–this selective politics of visibility. He identifies four different approaches to digital mapping, each with their own ethical challenges and opportunities. While aggregated approaches, such as the UN‐Habitat's mapping of areas against definitions of “slums,” can make people's needs in these areas visible, the abstract measures used often gloss over–and may miss–important aspects of the lived experience of living in deprived urban areas. Non‐governmental Organisations (NGO)‐led community mapping projects such as Slum Dwellers International or the Humanitarian OpenStreetMap Team can enable inhabitants themselves to map their spaces in ways that capture data that matter to them, but they are not always considered in state census efforts. The third and fourth approaches combine public and commercial satellite and drone imagery with semi‐automatic image classification approaches to categorize city spaces based on the degree of building density and layout patterns, again enhancing visibility, but often based on highly selective and opaque algorithmic and artificial intelligence‐based classification mechanisms.

Oluoch stresses that digital maps and charts comparing the rate of infections in countries are turning bodies, cities, and countries into points of exposure in the fight against the virus. He shows that the integration of multiple different systems of mapping may allow governments to “do things at a distance,” without engaging with vulnerable and marginalized populations and how this may “cumulatively pose a threat to civil liberties.” However, community mapping also opens up opportunities for just and participatory governance of crises and empowered governmentality. In this landscape, the ethical costs of using geo‐data‐driven decisions must be taken into account and Oluoch argues that community mapping projects can help “make the invisible” visible and lend “moral weight” to geoinformation as a method for empowerment and resilience.

Finally, the paper by Rosamunde van Brakel, Olya Kudina, Chiara Fonio, and Kees Boersma draws on socio‐technical perspectives and theories by analysing the technical, social, and institutional dimensions of two contact tracing apps developed and used in the Netherlands (CoronaMelder) and in Belgium (Coronalert) in response to the COVID‐19 crisis. They examine efforts to embed attention to digital ethics in the design of these track and trace applications. Discussing the use of ethics‐as‐accompaniment approaches (Verbeek, 2011) and citizen panels (Verbeek, 2020), the article shows that technical readiness is not sufficient to promote trust, social acceptance, or acceptability of surveillance measures, such as contact tracing apps.

Van Brakel et al. show that even when ethics is a prominent concern throughout the design process, acceptance may stay low, and unintended negative consequences can arise. For example, in both examples from the Netherlands and Belgium, great emphasis was placed on privacy protection and voluntary uptake. This was important “to prevent societal division and to ensure that nobody would be discriminated against if they chose not to use” the track and trace app. This stands in stark contrast to the way in which the uptake of health code apps in China was also “not enforced individually, but without it, living in the pandemic was severely restricted”–a form of compulsion by stealth (Bernot and Siqueira Cassiano, this issue) that is very common in surveillance capitalism worldwide. While the more genuinely voluntary approach in the Netherlands and Belgium reassured prospective users, it also compromised public commitment to collective responsibility and solidarity. The authors show that citizen panel members recognized and complained that, as a result of pitting privacy versus solidarity, people did ‘not see the point’ of the app, undermining its usefulness. Van Brakel et al. propose that a better understanding of the moral and socio‐ethical landscape is key for bridging values and finding the right balance between privacy and control. But they also show that more than attention to digital ethics is needed.

Van Brakel et al. identify the perhaps biggest obstacle to this effort. When the Dutch government attempted to engage with issues raised in the citizen panels and open up the development process of their app, the “proprietary hidden nature of the Google/Apple API, the core aspect of CoronaMelder, tainted these efforts” and raised long‐standing questions about the power of platform corporations. Morozov, Bria, and Wu's work is highly relevant here, opening new avenues for future research.

Overall, this special issue develops novel insight at the intersection of crisis management, surveillance studies, and digital ethics research. The convergence of surveillance society, surveillance, and disaster capitalism has accelerated in the wake of the ongoing COVID pandemic and is highly likely to deepen and broaden in the unfolding climate emergency. We argue that joining forces at this interdisciplinary juncture could be very powerful. Such a joining can provide novel analytical and practical policy traction. It allows a deeper and clearer understanding of intended and unintended ethical and political consequences, such as a politics of visibility that makes the operation of power invisible and fails to combat inequality, whilst ignoring the potentially positive power of digital data and surveillance for empowerment and resilience. By bringing different disciplines and different perspectives from different countries into dialog, we can strengthen responsible and circumspect socio‐technical innovation for crisis governance.

## Data Availability

The data that support the findings of this study (i.e., the article *Crisis management, surveillance, and digital ethics in the COVID‐19 era*) are available from the corresponding author, F. K. Boersma, upon reasonable request.

## ENDNOTE

1 NBC News 25 October 2021 https://www.nbcnews.com/tech/tech-news/facebook-whistleblower-documents-detail-deep-look-facebook-rcna3580
